# Outcomes of Microscopic vs. Endoscopic Tympanoplasty at a Tertiary Healthcare Institution in Western Maharashtra

**DOI:** 10.7759/cureus.70833

**Published:** 2024-10-04

**Authors:** Sweta Colvin, Abhay D Havle, Swapna A Shedge, Ganesh Vihapure, Aditya M P, Samiksha Karna, Shafeem Khan

**Affiliations:** 1 ENT, Krishna Vishwa Vidyapeeth (Deemed to be University), Karad, IND

**Keywords:** chronic suppurative otitis media (csom), endoscopic, graft uptake, hearing outcome, microscopic, tympanoplasty

## Abstract

Introduction

Chronic suppurative otitis media (CSOM) involves tympanic membrane perforation and is traditionally treated with microscopic tympanoplasty. Recent advancements in endoscopic techniques offer enhanced visibility and outcomes. This retrospective study compares endoscopic and microscopic tympanoplasty outcomes in CSOM patients between 2023 and 2024.

Aim

This study aims to evaluate outcomes of endoscopic vs. microscopic tympanoplasty in patients suffering from CSOM who are presenting at our tertiary care center.

Methodology

A retrospective study of 100 patients of CSOM undergoing tympanoplasty. Sixty patients underwent microscopic tympanoplasty and 40 endoscopic tympanoplasty. Postoperative successful uptake of graft and resultant hearing were compared between the two groups.

Results

Ninety-seven out of 100 patients had a successful graft uptake at three months. Fifty-eight patients in the microscopic tympanoplasty group (96.67%) and 39 in the endoscopic group (97.5%) had healed the tympanic membrane at three months of follow-up (p = 0.864). At postoperative three months, no statistically significant difference was found in the improvements in air conduction levels of the two groups (p = 0.995). No significant difference in the air-bone gap between the two groups (p = 0.095). The average air-bone gap at three months postoperative was 7.95 ± 4.20 decibel (dB) in the microscopic tympanoplasty group and 7.80 ± 4.10 dB in the endoscopic tympanoplasty group (p = 0.680). The incidence of postoperative wound area pain, numbness, and ear discharge was significantly higher in patients undergoing microscopic tympanoplasty (p < 0.05).

Conclusion

The two methods did not significantly differ in terms of the outcome of the hearing test or the rate of graft uptake. However, a better result was noted in endoscopic tympanoplasty compared to the microscopic tympanoplasty group. Postoperative complications encountered were less in the endoscopic tympanoplasty group.

## Introduction

Chronic suppurative otitis media (CSOM) is a middle ear disease characterized by perforated tympanic membrane (TM) [1]. The patients’ primary complaints are ear pain and hearing loss [2]. The postauricular approach with conventional microscopic tympanoplasty is the gold standard surgery for the repair of perforated TM and hearing loss improvement in CSOM [3,4]. Endoscopes’ role in otology has evolved during the last few decades [5]. With the advent of endoscopic ear instruments, endoscopic ear surgery has been more feasible and successful in recent years [6]. Unlike a microscope, an endoscope has a cone-shaped source of illumination, which permits better visibility and greater field of vision [7]. Endoscope-assisted microscopic ear surgery has developed since McKennan introduced endoscopy for second-look ear surgery in 1993, and Thomassin et al. used endoscopy along with microscope in ear surgery in 1993 [8]. McKennan, in 1993, introduced endoscopy for second-look ear surgery [9]. Thomassin et al. cleared residual cholesteatoma in sinus tympani and retro tympanum using 30- and 70-degree endoscopes [10]. In this retrospective analysis, we compared the preoperative audiometry results and symptoms with postoperative graft uptake and improvement in hearing in patients who underwent endoscopic vs. microscopic tympanoplasty for CSOM between 2023 and 2024.

## Materials and methods

This retrospective study was done to determine the functional outcome of tympanoplasty in patients with CSOM at Krishna Institute of Medical Sciences and Hospital, Krishna Vishwa Vidyapeeth, between January 2023 and January 2024. The study was reviewed by the Institutional Review Board of the Krishna Institute of Medical Sciences and Hospital, Krishna Vishwa Vidyapeeth.

A total of 112 underwent tympanoplasty during this period. The data were collected by following up on the medical charts and recording them in the Excel sheet (Microsoft® Corp., Redmond, WA). The study group included patients with TM perforation, dry ears for four weeks with intact ossicular chains, and normal middle ear mucosa. We excluded patients less than 18 years of age, those with sensorineural hearing loss, those requiring ossiculoplasty or mastoidectomy, and those with a previous history of ear surgery. This study covered 100 patients in total, as 12 patients out of 112 were lost to follow-up. Patients were divided into two groups; one underwent endoscopic tympanoplasty, and another group received conventional microscopic tympanoplasty. Depending on the patients’ needs, either local or general anesthesia was used throughout the procedure. Preoperative and postoperative assessments were done and documented in charts. The data was reviewed through medical records, and an analysis was done. The parameters compared were successful graft uptake, postoperative complications, and their preoperative and postoperative air conduction, pure-tone average (PTA), bone conduction PTA, and air-bone gap (ABG).

Surgery was done under local anesthesia based on the patients’ fitness. Patients in the microscopic group underwent postauricular approach tympanoplasty, and a temporalis fascia graft was harvested, which was placed using the underlay technique medial to remnant TM. Gelfoam (Pfizer Inc., New York, NY) was used to fill the external auditory canal (EAC) and middle ear spaces. In the endoscopic group, a tympanoplasty temporalis fascia graft was harvested from the incision over the incisura terminalis. The graft was placed using the underlay technique medial to the TM.

The main outcome of this retrospective study, being a surgical outcome, included graft uptake rate and postoperative complications, if any. Hearing outcomes included preoperative and postoperative air-bone gap and improvement. EAC Gelfoam was cleared by three weeks. The patient received microscopic and audiometric assessment at three months. A successful graft uptake was said to be a fully healed grafted TM without any perforation and retraction at three months postoperatively.

Preoperative and postoperative air-bone conduction audiograms at each of the four tested frequencies (500, 1,000, 2,000, and 4,000 Hz) were documented in the audiometric data. The ABG was determined using these data. Postoperative results were calculated using the audiogram done at three months.

Statistical analysis

SPSS version 27.0 (IBM Corp., Armonk, NY) was used for data analysis. The t-test for independent samples and the chi-square test were employed in the statistical analysis. A p-value of less than 0.05 was considered statistically significant.

## Results

Out of 100 patients, 60 (32 males, 28 females) had undergone microscopic tympanoplasty, while 40 (22 males, 18 females) underwent endoscopic tympanoplasty.

The mean age in the microscopic tympanoplasty group was 38.89 ± 11.6 years as compared to 41.23 ± 11.2 years in the endoscopic tympanoplasty group. The mean age difference was not statistically significant (Table 1).

**Table 1:** Clinical and demographics of patients in two groups

		Microscopic tympanoplasty group (n = 60)	Endoscopic tympanoplasty group (n = 40)
Average age ± standard deviation (years)		41.23 ± 11.2	38.89 ± 11.6
Gender	Male	32	22
	Female	28	18
Approach	Transcanal	0	40
	Postauricular	60	0
Graft material: temporalis fascia muscle		60	40

After surgery, every patient was monitored for three months. The successful graft uptake rate at three months in the microscopic tympanoplasty group was 58 (96.67%) as compared to 39 (97.5%) in the endoscopic tympanoplasty group. The differences between the two studies in graft uptake, however, did not reach statistical significance (p = 0.864).

The group that had microscopic tympanoplasty saw a significantly greater incidence of postoperative problems (p < 0.05). However, there were no appreciable variations seen in altered taste and tinnitus. Facial weakness and profound hearing loss were not encountered postoperatively (Table 2).

**Table 2:** Surgical outcomes at three-month follow-up The chi-square test was used to calculate the p-value.

	Microscopic tympanoplasty group (n = 60)	Endoscopic tympanoplasty group (n = 40)	p
Graft uptake	58 (96.67%)	39 (97.5%)	0.864
Postoperative complications			
Ear pain	49 (81.67%)	4 (10%)	<0.05
Numbness around the ears	11 (18.33%)	0	<0.05
Discharging ears	2 (3.33%)	0	0.045

Preoperative air conduction of the operative ear in the microscopic tympanoplasty group was 36.75 ± 9.90 decibel (dB) and 38.12 ± 9.30 dB in the endoscopic tympanoplasty group, which had no significant statistical difference (p = 0.205). ABG in the two groups were 18.20 ± 6.00 dB and 19.25 ± 6.40 dB, respectively, without a difference that is statistically significant (p = 0.115).

Postoperative audiological assessment done at three months showed 26.05 ± 7.30 dB improvement in air conduction in the microscopic tympanoplasty as compared to 26.10 ± 7.25 dB in endoscopic tympanoplasty, which was not statistically significant (p = 0.995). There was no change in bone conduction noted postoperatively in both groups. Postoperative ABG at three months follow-up in each group were 7.95 ± 4.20 dB and 7.80 ± 4.10 dB, respectively (p = 0.680).

The endoscopic tympanoplasty group showed an improvement in ABG closure of 11.80 ± 5.40 dB, more than 10.75 ± 5.30 dB seen in the microscopic tympanoplasty group. There were no statistically significant differences in the ABG improvement between the two groups at three months postoperatively (p = 0.095) (Table 3).

**Table 3:** Preoperative and postoperative (at three-month follow-up) hearing outcomes Unpaired t-test has been used for the p-value.

	Microscopic tympanoplasty group (n = 60) dB	Endoscopic tympanoplasty group (n = 40) dB	p
Air conduction			
Preoperative	36.75 ± 9.90	38.12 ± 9.30	0.205
Postoperative	26.05 ± 7.30	26.10 ± 7.25	0.995
Bone conduction			
Preoperative	18.40 ± 5.20	18.70 ± 1.65	0.590
Postoperative	18.26 ± 4.75	18.28 ± 4.60	0.940
Air-bone gap			
Preoperative	18.20 ± 6.00	19.25 ± 6.40	0.115
Postoperative	7.95 ± 4.20	7.80 ± 4.10	0.680
Air-bone gap closure			
Postoperative	10.75 ± 5.30	11.80 ± 5.40	0.095

## Discussion

The main objectives of tympanoplasty are to provide a healthy tympanic cavity, close the TM perforation, and improve hearing as much as possible [11]. The postauricular approach with microscopic tympanoplasty is the traditional and most widely applied technique for tympanoplasty. Success rates noted in various studies are ranging from 83% to 100% [12-15]. However, surgeons’ vision is restricted due to the linear vision of the microscope [16]. Resulting in soft tissue dissection and retraction with bone drilling to adequately see the affected area [17]. This may lead to improper disease clearance, inadequate graft placement, and higher chances of postoperative complications [18]. Patients in our study who underwent microscopic tympanoplasty had more complaints of postoperative pain, numbness, and discharging ears (p < 0.000). Various studies by Hsu et al. and Choi et al. noted that patients undergoing endoscopic tympanoplasty had significantly lesser postoperative complications such as postoperative pain, persistent otorrhea, and numbness [6,13]. Endoscopic tympanoplasty provides good light exposure and a better viewing angle of all the hidden areas seen under a microscope [19,20]. Endoscopic tympanoplasty creates an operational region in EAC, resulting in no hair loss, very minimal soft tissue injury, and a small wound for graft harvesting [21-23].

In our study, the effective graft uptake in the group undergoing microscopic tympanoplasty was 58 out of 60, whereas the group undergoing endoscopic tympanoplasty had a comparable success rate of 39 out of 40. According to Yang et al., there was no discernible difference between endoscopic and microscopic groups (97.50% vs. 97.77%). Hsu et al. also noted similar graft uptake rates in the two groups (96.2% vs. 92%) [6]. A meta-analysis done by Tseng et al. in 2016, including four studies, suggested a comparable TM perforation closure rate in both groups. This study suggested the success rate of perforation closure was related to the grafting techniques, i.e., underlay technique, as compared to the different approaches [24].

In our study, both groups had similar postoperative hearing improvement and ABG closure. There were no statistical differences in preoperative ABG (p = 0.115) and postoperative ABG at three months (p = 0.680) in the microscopic and endoscopic groups. The average postoperative air-bone gap closure at three months in both groups was similar (p = 0.095). The average postoperative air-bone gap closure in microscopic vs. endoscopic tympanoplasty groups were 10.27 ± 6.4 dB and 12.43 ± 7.46 dB, respectively, according to Hsu et al. (p = 0.166) [6].

The average ABG closure in the present study was 10.75 ± 5.30 in the microscopic tympanoplasty group, which was similar to different studies on microscopic tympanoplasty (Table 4) [13,14,25,26].

**Table 4:** Comparison of average air-bone gap closure in microscopic tympanoplasty with different studies

Study	Average air-bone gap closure (dB)
Present study	10.75 ± 5.30
Zakir et al. [25]	10.98 ± 4.21
Choi et al. [13]	6.1 ± 1.0
Lee et al. [26]	5.27 ± 6.90
Yang et al. [14]	10.48 ± 5.18

The average ABG closure in our study was 11.80 ± 5.40 in the endoscopic tympanoplasty group, which was comparable to various studies on endoscopic tympanoplasty (Table 5) [24,27,28]. 

**Table 5:** Comparison of average air-bone gap closure in endoscopic tympanoplasty with different studies

Study	Average air-bone gap closure (dB)
Present study	11.80 ± 5.40
Tadke et al. [27]	11.46 ± 1.13
Tseng et al. [24]	10.3
Raj et al. [28]	8

Limitations

The study was constrained by a limited time frame and a small patient sample, necessitating a larger and more comprehensive study in the future. Being a retrospective study, future research should consider a prospective design to validate and expand upon these findings. Also, due to the retrospective nature of the study, an evaluation of various graft materials was not feasible. Future studies should incorporate diverse graft types to assess their relative effectiveness. This study did not explore different surgical approaches with a microscope, such as the endaural approach. Investigating these alternative approaches could provide additional insight. With the lack of an endoscope holder affecting the ability to use dual-hand techniques, future research should address this limitation to enhance procedural effectiveness.

## Conclusions

Both techniques have similar hearing improvement postoperatively. Also, the two methods did not significantly differ in terms of the rate of graft uptake. However, the endoscopic tympanoplasty group had better outcomes in view of postoperative ear pain, numbness, and discharging ear. Thus, we conclude that an endoscopic tympanoplasty may be preferred when performing tympanoplasty.
